# Supplementary material for: Persistent Post-COVID-19 Syndrome in Hemodialyzed Patients—A Longitudinal Cohort Study from the North of Poland
Source: J Clin Med. 2021 Sep 28;10(19):4451. doi: 10.3390/jcm10194451 (PMC8509624; doi:10.3390/jcm10194451)
Supplement: Supplementary file 1 [file jcm-10-04451-s001.zip › jcm-1355201-supplementary.pdf]

Form S1. Symptom Questionnaire (SRSQ)

1. How would you comment your current health status?

□ Same as prior to COVID-19 □ Often feel fatigue, and easier to get tired after activity now than prior to COVID-19 □ Better health condition than prior to COVID-19

2. Have you experienced any of the following pain symptoms that appeared post COVID-19 and are persistent?

□ No □ Headache □ Myalgia □ Chest pain □ Joint pain (if yes, please fill in the table below)

□ Any other if yes, please specify…………

Joints	Hand	Foot	Wrist	Ankle	Jaw	Elbow	Shoulder	Neck	Hip	Knee
Tenderness										
Swollen										
Numerical pain scale (0-10)*										

*0-No pain; 10-Intolerable pain

3. Do you have any of the following symptoms that are newly onset post COVID-19 and persistent?

□ No □ Sore throat □ Difficult to swallow

4. Do you have any of the following symptoms that are newly onset post COVID-19 and persistent?

□ No □ Low grade fever (37.3-38.0°C) □ Palpitations □ Dizziness □ Nasal congestion □ Skin rash

5. Are you more prone to suffer from the following symptoms after discharge?

□ No □ Diarrhea □ Nausea □ Vomiting

6. How do you feel about your sense of smell compared with the status prior to COVID-19?

□ Same as before □ Worse than before □ Better than before □ Total loss

7. How do you feel about your sense of taste compared with the status prior to COVID-19?

□ Same as before □ Worse than before □ Better than before □ Total loss

8. How do you feel about your appetite compared with the status prior to COVID-19?

□ Same as before □ Worse than before □ Better than before

9. What do you think about your sleeping compared with the status prior to COVID-19?

□ Same as before □ Worse than before □ Better than before

10. How do you feel about your muscle strength compared with the status prior to COVID-19?

□ Same as before □ Worse than before □ Better than before

11. Have you experienced hair loss now compared with the status prior to COVID-19?

□ No hair loss before or after COVID-19 □ Hair loss is same as before □ Lose more hair than before
□ Lose less hair than before
